# The Role of Pathogens in Bumblebee Decline: A Review

**DOI:** 10.3390/pathogens14010094

**Published:** 2025-01-18

**Authors:** Huanhuan Chen, Nawaz Haider Bashir, Qiang Li, Chao Liu, Muhammad Naeem, Haohan Wang, Wenrong Gao, Richard T. Corlett, Cong Liu, Mayra C. Vidal

**Affiliations:** 1 College of Biological Resource and Food Engineering, Qujing Normal University, Qujing 655011, China; chenhuanhuan@caas.cn (H.C.); nawazhaider@caf.ac.cn (N.H.B.); lq@mail.qjnu.edu.cn (Q.L.); naeem@mail.qjnu.edu.cn (M.N.); wanghh@eastern-himalaya.cn (H.W.); gaowenrong2012@163.com (W.G.); 2 Key Laboratory of Yunnan Provincial Department of Education of the Deep-Time Evolution on Biodiversity from the Origin of the Pearl River, Qujing Normal University, Qujing, 655011, China; 3 Key Laboratory of Insect-Pollinator Biology of Ministry of Agriculture and Rural Affairs, Institute of Apicultural Research, Chinese Academy of Agricultural Sciences, Beijing 100193, China; 4 Center for Integrative Conservation, Xishuangbanna Tropical Botanical Garden, Chinese Academy of Sciences, Mengla 666303, China; corlett@xtbg.org.cn; 5 Biology Department, University of Massachusetts Boston, Boston, MA 02125, USA; mayra.cadorinvidal@umb.edu; 6 Department of Organismic and Evolutional Biology, Museum of Comparative Zoology, Harvard University, Cambridge, MA 02138, USA

**Keywords:** *Bombus* species, bee decline, *Vairimorpha*, *Crithidia*, viruses, pathogens

## Abstract

Bumblebees, the most important wild pollinators in both agricultural and natural ecosystems, are declining worldwide. The global decline of bumblebees may threaten biodiversity, pollination services, and, ultimately, agricultural productivity. Several factors, including pesticide usage, climate change, habitat loss, and species invasion, have been documented in the decline of bumblebee species, but recent studies have revealed the dominating role of pathogens and parasites over any of these causes. Unfortunately, there is a lack of a full understanding of the role of pathogens and parasites in the decline of bumblebee species. The current study provides a comprehensive review of how pathogens and parasites contribute to the decline of bumblebee species. The study also explores the prevalence of each pathogen and parasite within bumblebee populations. Furthermore, we address the synergistic effects of pathogens and other stressors, such as pesticides, climatic effects, and habitat loss, on bumblebee populations. To summarize, we propose possible conservation and management strategies to preserve the critical role of bumblebees in pollination services and thus to support ecosystem and agricultural health.

## 1. Introduction

Bumblebee insect species belong to the genus *Bombus* (Apidae: Hymenoptera), which contains 250 species in sub-tropical, sub-temperate, and temperate regions with bright colors and large sizes [1,2]. Bumblebees, like other bees, are phytophagous, sustaining themselves for their entire lives on nectar and pollen [3]. Their young ones are sedentary, so adult female bumblebees facilitate them by gathering their food [4]. The mouthparts consist of a proboscis for sucking nectar, while the hind legs are modified for pollen collection [5]. *Bombus* species exhibit a high abundance and frequent flower visitation, particularly during the spring and summer seasons, predominantly in temperate environments [6]. They are one of the most effective natural pollinators of food crops and plants because of their ability to “buzz” pollinate and fly in cool temperatures [7,8,9]. Buzz pollination refers to a vibration produced by bees using their thoracic muscles to extract the pollens from flowers [10]. *Bombus* species such as *Bombus terrestris* Linnaeus, *B. occidentalis* Greene, *B. lucorum* Linnaeus, *B. impatiens* Cresson, and *B. ignitus* Smith [11,12,13] have been utilized commercially in greenhouses since the 1980s to pollinate strawberries, blueberries, eggplants, vegetables, and tomatoes, as well as fruit trees [11]. Bumblebees are responsible for providing the most substantial quantitative pollination services to agriculture, leading to enhanced profitability, increased crop yield, and reduced labor expenditures [9,14]. With the exception of Africa and Antarctica, bumblebees are currently reared for commercial purposes on all other continents, with the annual global sales volume reaching 1,000,000 colonies [15]. The bumblebee market has evolved from its initial concentration on greenhouse pollination services to now include the open-field pollination of crops that are visited by bumblebees [11]. There are currently over 60 countries where bumblebees are used commercially for pollination [16], and this number is expanding rapidly [17].

**Figure 1:**
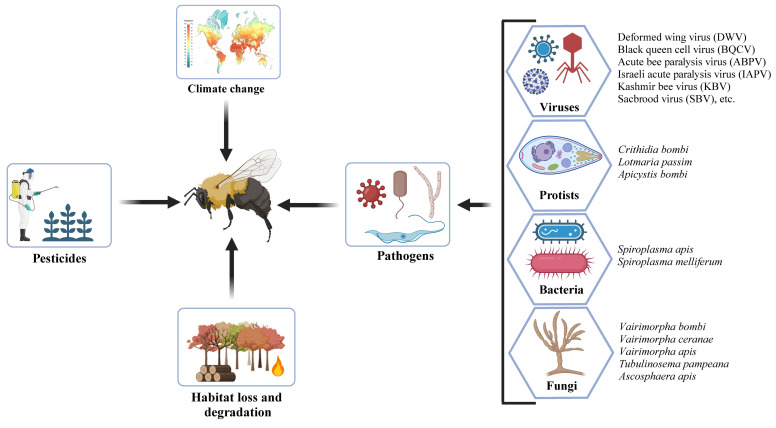
Factors affecting bumblebees’ health (references in the text).

Nevertheless, numerous species of bumblebees are currently experiencing significant population decreases [18,19], and this has major implications for food security, agriculture, and essential ecological services [20,21]. Over the last decade, the evidence for this decline has become increasingly pronounced, with notable reductions in both the distribution and relative abundance of these pollinators across North America [18,22,23,24], South America [25,26], Europe [19,27,28], and Asia [29]. This global decline of bumblebees has been attributed to several biotic and abiotic factors [30], such as habitat fragmentation, floral diversity reduction, exposure to pesticides, climate change, invasive species, and the prevalence of pathogens and parasites (illustrated in Figure 1), highlighting the urgent need for comprehensive conservation strategies [31,32].

Pathogens, including protists, bacteria, viruses, and fungi, play a critical role in the decline of bumblebee populations (Figure 1) [33,34]. These pathogens can cause diseases that not only affect individual bumblebees’ fitness but also have devastating impacts on entire colonies, leading to population decreases [35]. Several transmission modes have been identified, including horizontal transmission, which involves interactions within the same environment or direct bee-to-bee contact, and vertical transmission, which occurs when a mother passes pathogens to her offspring [36]. The dense hive monocultures during rearing in greenhouses could potentially spread diseases among bumblebees [37]. A new concern is arising from the trade and movement of managed bees, which facilitate the spread of pathogens from bees to new hosts (e.g., bumblebees), a phenomenon known as spillover [38,39]. For example, the deformed wing virus (DWV) has been found in bumblebees that share habitats with honey bees [40,41,42,43].

In this review, we summarize and discuss the impact of pathogens on bumblebee populations. Based on the existing literature, we provide a comprehensive overview of the major pathogens and their role in bumblebee decline. We assess the significance of the species and host and discuss the known effects of pathogens on bumblebee health and colony dynamics. Furthermore, we highlight the research gaps and limitations regarding bumblebee pathogens and suggest future research directions. Finally, we explore the synergistic effects that pathogens and other stressors contribute to bumblebee decline and suggest management strategies to mitigate the impact of pathogens on bumblebee populations.

## 2. Methodology

**Figure 2:**
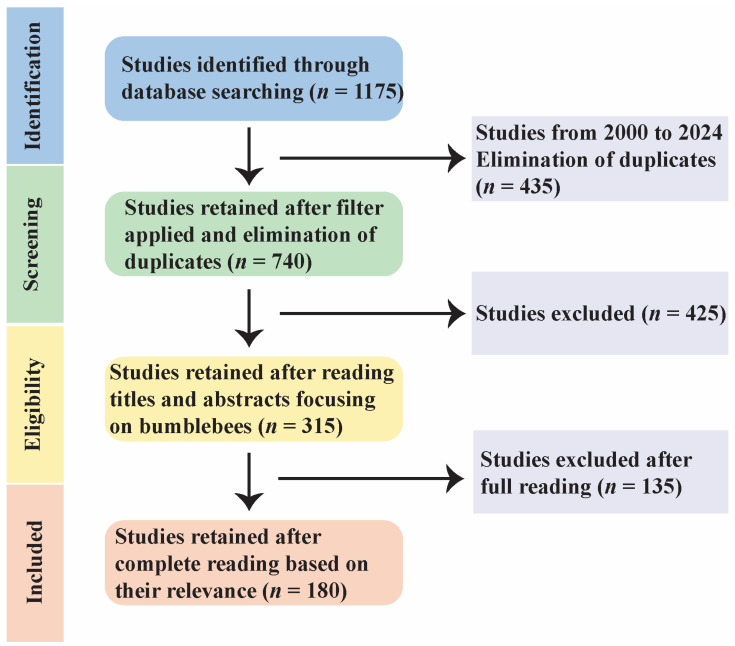
Flowchart of the literature search and screening process in this study.

For the literature review, bumblebee species were considered. We used the Google Scholar, Web of Science, and PubMed search engines by using the keywords “bumblebee”, “pathogens”, “parasites”, and “population decline”. We included empirical studies, meta-analyses, and bumblebee pathogens related to species decline (Figure 2).

## 3. Important Bumblebee Pathogens

### 3.1. Overview of Bumblebee Pathogens

**Figure 3:**
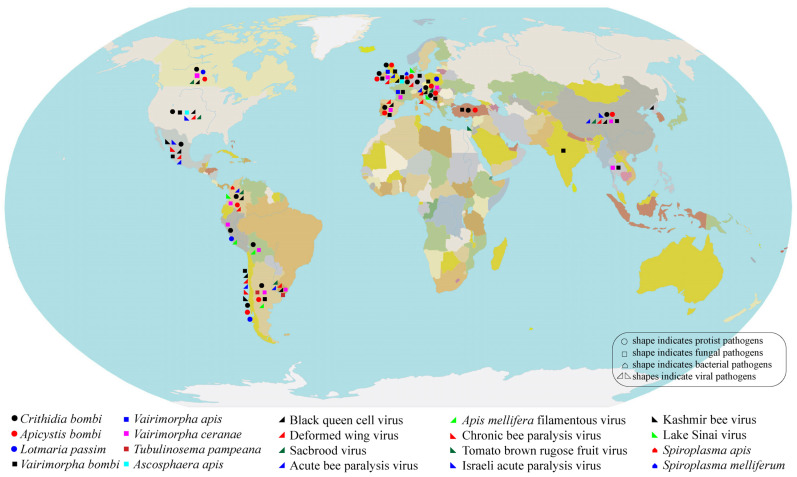
Distribution and prevalence of bumblebee pathogens (points represent the country-level prevalence of pathogens, not the exact locations of pathogens).

Pathogens have a deleterious impact on the health, abundance, and population numbers of bumblebees, contributing to the population decline [44]. Upon reviewing the literature, we found 85 reports describing 20 pathogens associated with bumblebees across 25 countries (Figure 3, Table 1 and Table 2). Trypanosome and microsporidian parasites, such as *Crithidia bombi* and *Vairimorpha* (*Nosema) bombi*, affect the hindgut and digestive systems of bumblebees. This reduces their foraging capacity, compromises colony success, and lowers their overall fitness [45,46,47]. Viruses like the deformed wing virus (DWV) can cause morphological abnormalities, a shortened lifespan, and decreased foraging effectiveness [48]. Larval mortality is also influenced by fungal infections such as chalkbrood disease [49] and bacterial pathogens like *Spiroplasma* [50].

#### 3.1.1. Viruses

Infection with different viruses can contribute to the documented decline in pollinator diversity and density worldwide [51]. Various viral infections threaten bees; among them, the most studied are the black queen cell virus (BQCV), the acute bee paralysis virus (ABPV), the sacbrood bee virus (SBV), the chronic bee paralysis virus (CBPV), the Kashmir bee virus (KBV), and the deformed wing virus (DWV) [39,52]. *Varroa destructor*, a common mite that feeds on various stages of adult bees in colonies, is known as an active vector of these viruses [53]. Many studies have focused on bee pathogenic viruses to understand the correlation between bee losses and their effect on bee health [54]. The significant pollinators, bumblebees, are likewise threatened by a number of pathogens affecting honey bees and wild pollinator species [40,55].

**Figure 4:**
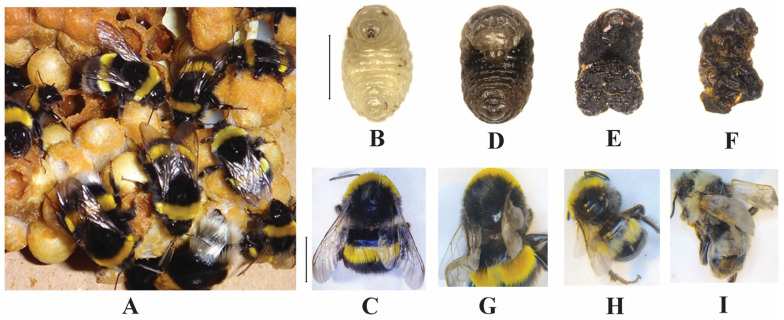
*Bombus terrestris*: (**A**) healthy colony, (**B**) healthy fourth instar larva, (**C**) healthy adult, (**D**–**F**) *Ascosphaera apis* chalkbrood-infected fourth instar larvae, (**G**–**I**) deformed wing virus infected adults; scale bars = 50 mm (**B**,**D**–**F**), 5 mm (**C**,**G**–**H**) (sources: Zhang et al. [78] (**A**), Pereira et al. [16] (**B**,**D**–**F**), and Cilia et al. [15] (**C**,**G**–**I**)).

The deformed wing virus (DWV), one of the most prevalent honey bee pathogens, has been found in other insect species, including *Bombus* adults from both managed and wild colonies (Figure 4) [15,42,48]. DWV is a positive-sense ssRNA virus belonging to the Iflaviridae family and the *Iflavirus* genus [56,57,58]. DWV is currently recognized as having three genetic variants: type A, type B, and type C, with type A and B being the most common in bees [59,60,61]. The parasitism of *V. destructor* is mostly linked to DWV, and this combination poses the most significant health threat to bee populations [62,63]. DWV infections have been reported in *B. atratus* [64,65], *B. ephippiatus* [66], *B. humilis* [51], *B. huntii* [67], *B. impatiens* [17], *B. lapidarius* [40,51], *B. lucorum* [51], *B. pascuorum* [42,68,69,70,71], *B. pauloensis* [39], *B. ruderatus* [72], *B. steindachneri* [66], *B. ternarius* [41], *B. terrestris* [16,40,51,68,69,70,71,72], and *B. vagans* [41,43] (Table 1). DWV can be transmitted from infected honey bees to bumblebees through shared flowers and can even be deposited on floral surfaces by bumblebees that have consumed only sterile sucrose after 72 hours [73]. The presence of DWV in pollen pellets and bee feces suggests that honey bee foragers, through their foraging activities, and colonies, by acting as reservoirs of high viral loads, may facilitate the horizontal transmission of this virus to the broader pollinator community [41,74]. DWV is transmitted horizontally by bee-to-bee contact and the consumption of contaminated food [15,75], and it can also spread within a colony through the puncture of the cuticle in both the juvenile and adult stages [76,77]. Longer foraging times increase virus acquisition in bumblebees, making foraging time a key factor in DWV disease dynamics [73].

The black queen cell virus (BQCV) belongs to the genus *Triatovirus* within the Dicistroviridae family and is part of the Picornavirales order [79,80]. This virus is known to affect several species of bees, including various *Apis* and bumblebee species, acting as the etiological agent of fatal diseases in those insects [81]. For example, BQCV infections have been documented in numerous bumblebee species such as *B. terricola* [21], *B. pauloensis* [39], *B. hortorum, B. lapidarius*, *B. sylvarum, B. terrestris, B. pascuorum*, *B. humilis* [51], *B. ephippiatus*, *B. steindachneri* [66], *B. bimaculatus*, and *B. vagans* [82]. It has been demonstrated by Tsvetkov et al. [21] that *B. terricola* collected from the agricultural environment shows a higher prevalence of BQCV, notably within worker bees. Moreover, the Israeli acute paralysis virus (IAPV), belonging to the same Dicistroviridae family as BQCV [83], was initially discovered in the hives of infected *Apis mellifera* [84] and later found in other pollinators, including several bumblebee species [41]. Three such species, *B. ternarius, B. impatiens,* and *B. vagans,* have been positively identified as hosts for IAPV [17,41]. IAPV may be transmitted from honey bees to bumblebees; in vitro transfer of this virus resulted in pseudo-queens [85]. The sacbrood virus (SBV) has separately been reported at varying prevalences within wild pollinators and honey bees, having the capacity to infect brood and adult hosts [86]. The Lake Sinai virus (LSV) is a single-stranded positive RNA virus that encompasses three key genes: non-structural protein, capsid protein, and RNA-dependent RNA polymerase [87]. The virus could impair the immune system of the host organism, increasing its susceptibility to other stressors and pathogens [88]. Recently, LSV has also been detected in certain bumblebee species (Table 1).

**Table 1:** Virus prevalence reported from bumblebee species.

Bumblebee Species	BQCV	DWV	SBV	ABPV	AmFV	CBPV	KBV	LSV	ToBRFV	IAPV
*B. armeniacus*	√									
*B. atratus*	√	√	√	√				√		
*B. bimaculatus*	√	√								
*B. bohemicus*				√						
*B. braccatus*	√		√							
*B. breviceps*	√									
*B. convexus*	√									
*B. cryptarum*	√			√						
*B. cullumanus*	√									
*B. dahlbomii*	√			√		√	√			
*B. ephippiatus*	√	√								
*B. friseanus*	√									
*B. funebris*					√					
*B. jonellus*				√						
*B. hortorum*	√		√	√				√		
*B. humilis*	√	√								
*B. huntii*	√									
*B. ignitus*	√									
*B. impatiens*	√	√		√		√	√			√
*B. impetuosus*	√									
*B. keriensis*	√			√						
*B. ladakhensis*	√									
*B. lantschouensis*	√									
*B. lapidarius*	√	√		√				√		
*B. lepidus*	√									
*B. longipes*	√									
*B. lucorum*	√	√		√						
*B. monticola*		√		√						
*B. opifex*					√					
*B. pascuorum*	√	√	√	√				√		
*B. patagiatus*	√									
*B. pauloensis*	√	√	√	√	√					
*B. pratorum*				√				√		
*B. pyrosoma*	√		√							
*B. ruderatus*	√	√		√		√	√			
*B. rufofasciatus*	√									
*B. sichelii*	√									
*B. soroeensis*	√	√								
*B. steindachneri*		√								
*B. subterraneus*	√									
*B. supremus*	√									
*B. sushkini*	√									
*B. sylvarum*	√		√					√		
*B. ternarius*	√	√	√							√
*B. terrestris*	√	√	√	√		√	√	√	√	
*B. terricola*	√		√							
*B. trifasciatus*	√									
*B. turkestanicus*	√									
*B. vagans*	√	√	√							√
*B. waltoni*	√									√

Note: BQCV = black queen cell virus [21,39,41,51,64,66,70,72,82,89,90,91,92,93,94,95,96,97,98], DWV = deformed wing virus [15,17,39,40,41,42,48,51,64,66,70,72,73,82,90,91,92,93,97,98,99,100,101,102,103,104,105], SBV = sacbrood virus [21,39,41,51,64,91,92], ABPV = acute bee paralysis virus [17,39,51,64,72,91,92,106,107], AmFV = *Apis mellifera* filamentous virus [44,108], CBPV = chronic bee paralysis virus [17,72], KBV = Kashmir bee virus [17,72], LSV = Lake Sinai virus [51,64,92,109], ToBRFV = tomato brown rugose fruit virus [110], IAPV = Israeli acute paralysis virus [17,41,91].

#### 3.1.2. Fungi and Protists

The microsporidian *Vairimorpha bombi*, formerly classified as *Nosema bombi,* belonging to the Microsporidia family, is an obligate fungal pathogen of bumblebees, affecting both natural and commercial populations worldwide [111,112]. *Vairimorpha bombi* has been found in various species of the *Bombus* genus, including *B. lapidarius, B. terrestris* [33], *B. haemorrhoidalis* [13,30], *B. montivagus*, *B. breviceps,* etc. (Table 2) [13]. Previous research has shown that *V. bombi* is more common in male individuals than in workers [113,114]. Both workers and larvae were found to be affected by *V. bombi* infection; however, the larval stage is more vulnerable [30,115]. The prevalence of *V. bombi* is higher in two declining species, *B. occidentalis* and *B. pensylvanicus,* than in other species [116]. Other intracellular microsporidian parasites, *Vairimorpha (Nosema) ceranae* and *Vairimorpha (Nosema) apis,* infect the host’s midgut epithelial cells [117,118]. *Vairimorpha ceranae* was originally detected in the Asian honey bee *Apis cerana* and is now widespread across the globe, recently being found in various species of bumblebees and solitary bees [119,120,121,122]. The frequency of *V. ceranae* has increased considerably in locations that are far from non-commercial bumblebee-utilizing greenhouse sites [104]. *Vairimorpha apis* has been detected in *B. terrestris* colonies near honey bee hives [104]. Recently, an emerging microsporidian pathogen called *Tubulinosema pampeana* was discovered in *B. atratus* from Argentina [123] and later in Uruguay from the infected tissues of the same species [124]. 

The *Ascosphaera* fungus (Eurotiomycetes: Ascosphaerales) is predominantly linked to bee larvae, and certain species of the fungus are harmful and induce chalkbrood disease in both social and solitary bee larvae [125,126]. This fungus is common in the environment [71], and wild bees can easily become infected via contaminated pollen while foraging on flowers. Maxfield-Taylor and co-workers [49] detected *Ascosphaera apis* in the queens of three species—*B. griseocollis*, *B. vosnesenskii*, and *B. nevadensis*—and found that the infected queens died within 21–121 days.

*Crithidia bombi* (Trypanosomatidae), an intestinal trypanosome, is a prevalent pathogen observed in bumblebees [33]. More than 50 bumblebee species have been reported to have *C. bombi* infections (Table 2). *Vairimorpha ceranae* and *C. bombi* have been observed to correlate with the decline of bumblebee populations in China [13]. The neogregarinid pathogen *Apicystis bombi* is a sporozoan that infects bumblebee species [127], and about thirty bumblebee species have been found to have *A. bombi* infections [16,33]. *Lotmaria passim* is a honey-bee-associated trypanosomatid recently detected in bumblebee species, including *B. dahlbomii, B. opifex, B. ruderatus, B. terrestris, B. pascuorum,* and *B. terricola*, from Canada, Chile, Poland, and Peru [21,44,72]. The transmission of *V. bombi* usually occurs when bees consume spores from contaminated food or during mating [128]. *Crithidia bombi* is primarily transmitted vertically through infected nest debris or nestmates, while horizontal transmission occurs through contaminated flowers and feces [33,129]. *Apicystis bombi* is transmitted when bees ingest oocysts, which develop into sporozoites in the gut and are eventually deposited in the fat body [130,131]. A recent study suggests that *A. bombi* oocysts may also be present in pollen batches obtained from honey bees [132]. Laboratory workers can contract parasites and viruses either from the queen or from the pollen provided to the larvae [39]. Because bumblebee colonies have an annual life cycle and only the queens survive the winter, the pathogens can continue to reproduce and spread after the colonies have disappeared [7,39].

#### 3.1.3. Bacteria

In general, the role of the bacteria reported from bumblebees is still unclear, whether beneficial or pathogenic, including *Bacillus pumilus*, *B. cereus*, *B. fusiformis*, *Spiroplasma melliferum*, *S. apis*, *Paenibacillus glucanolyticus*, *Enterobacter cloacae*, *Burkholderia cepacia*, and *Brevibacillus laterosporus* [64,133,134]. Among these, *S. melliferum* and *S. apis* bacteria are known to be pathogenic and linked to honey bee disease, which causes bee mortality [134]. Bacteria have also been identified on the flower surface and in the gut and hemolymph of pollinator insects, including bumblebees (Table 2) [64,134].

**Table 2:** Pathogen prevalence reported from bumblebee species.

Bumblebee Species	Protists			Fungi					Bacteria	
	CB	AB	LP	VB	VA	VC	TP	AA	SA	SM
*B. armeniacus*	√	√								
*B. atratus*	√	√				√	√		√	
*B. auricomus*				√						
*B. bellicosus*						√				
*B. bifarius*	√			√						
*B. bimaculatus*	√			√						
*B. braccatus*	√	√								
*B. breviceps*	√	√		√		√				
*B. californicus*				√						
*B. caliginosus*				√						
*B. centralis*	√			√						
*B. citrinus*				√						
*B. convexus*	√	√								
*B. cryptarum*	√	√		√						
*B. cullumanus*	√	√								
*B. dahlbomii*	√	√	√							
*B. fernaldae*				√						
*B. fervidus*	√			√						
*B. flavifrons*	√			√						
*B. frigidus*	√			√						
*B. friseanus*	√	√		√						
*B. funebris*	√									
*B. griseocollis*	√			√						
*B. haemorrhoidalis*				√		√				
*B. hortorum*	√	√		√						
*B. humilis*		√								
*B. huntii*	√			√						
*B. ignitus*	√	√								
*B. impetuosus*				√		√				
*B. impatiens*	√	√		√						
*B. insularis*				√						
*B. keriensis*	√	√								
*B. lantschouensis*	√	√								
*B. lapidarius*	√	√		√		√				
*B. lepidus*	√	√								
*B. longipes*	√	√								
*B. lucorum*	√	√		√						
*B. melanopygus*	√			√						
*B. mixtus*	√			√						
*B. montivagus*				√		√				
*B. morio*						√				
*B. nevadensis*								√		
*B. occidentalis*	√			√						
*B. opifex*	√		√			√				
*B. pascuorum*	√	√	√	√		√				√
*B. patagiatus*	√	√		√		√				
*B. pauloensis*	√						√			
*B. pensylvanicus*	√			√						
*B. perplexus*				√						
*B. pratorum*	√	√		√					√	
*B. pyrosoma*	√	√								
*B. remotus*						√				
*B. ruderatus*	√	√	√	√						
*B. rufocinctus*	√			√						
*B. rufofasciatus*	√	√		√						
*B. sibiricus*						√				
*B. sitkensis*				√						
*B. soroeensis*	√	√								
*B. subterraneus*	√	√								
*B. suckleyi*				√						
*B. sushkini*	√	√								
*B. sylvarum*	√	√								
*B. sylvicola*	√									
*B. ternarius*	√									
*B. terrestris*	√	√	√	√	√	√		√		
*B. terricola*	√		√	√		√				
*B. trifasciatus*	√	√								
*B. turkestanicus*	√	√								
*B. vagans*				√						
*B. vandykei*	√									
*B. vosnesenskii*	√			√				√		
*B. waltoni*	√	√				√				

Note: CB = *Crithidia bombi* [17,21,33,35,44,64,70,72,90,91,92,94,97,104,105,108,135,136,137,138,139,140,141,142,143,144,145,146,147,148], AB = *Apicystis bombi* [33,64,70,72,91,92,104,105,127,135,138,142,146,148,149], LP = *Lotmaria passim* [21,44,72,150], VB = *Vairimorpha bombi* [13,17,18,30,33,35,70,72,92,93,94,104,105,122,127,135,140,141,144,145,151,152,153,154], VA = *Vairimorpha apis* [104,152], VC = *Vairimorpha ceranae* [13,21,39,40,44,64,92,104,108,121,122,124,152,155,156], TP = *Tubulinosema pampeana* [39,123,124], AA = *Ascosphaera apis* [16,49], SA = *Spiroplasma apis* [64,134], and SM = *Spiroplasma melliferum* [134].

### 3.2. Impacts of Pathogens

Previous studies have shown that DWV infection in bumblebee species could cause wing malformations (Figure 4) and lead to colony loss, having a significant impact on the ecosystem supported by bumblebees [15,157]. Bumblebee larvae infected with BQCV develop a yellowish, sac-like integument and die [95]. IAPV infections have been observed to reduce significantly the lifespan of individual workers, posing a significant threat to the overall survival of bee colonies. Furthermore, IAPV infection impairs the navigational capacity of foraging workers, leading to their inability to return to the hive [158]. Paralysis symptoms, such as paralyzed front legs and severe body tremors, were observed in bumblebees after IAPV virus injection [159], and acute infection caused high mortality rates (Table 3) [160].

It has been shown that male bumblebees infected with *V. bombi* within colonies have a significantly reduced production of viable sperm, while infected females display swollen abdomens and a lack of willingness to mate, resulting in a decrease in the overall fitness of the colony [161]. Infected queens exhibit reduced colony sizes and produce fewer offspring, leading to population declines [46]. The infection propagates along the digestive tract and spores spread in the muscles, malpighian tubules, accessory glands, midgut, fat body, ovaries, and testes of reproductive adults [161]. In addition, *V. bombi* infections disturb the colony size and the lifespan of drones, workers, and queens (Table 3) [162,163]. *Vairimorpha ceranae* infects the gastrointestinal epithelial cells of adult bees, leading to negative effects on both the productivity and long-term survival of bee colonies [63,64,65,66,67,68,69,70,71,72,73,74,75,76,77,78,79,80,81,82,83,84,85,86,87,88,89,90,91,92,93,94,95,96,97,98,99,100,101,102,103,104,105,106,107,108,109,110,111,112,113,114,115,116,117,118,119,120,121,122,123,124,125,126,127,128,129,130,131,132,133,134,135,136,137,138,139,140,141,142,143,144,145,146,147,148,149,150,151,152,153,154,155,156,157,158,159,160,161,162,163,164,165]. A research study has shown that *V. ceranae* infection spreads from the midgut to other tissues and reduced the survival of bumblebees by 48% [155].

The parasite *Crithidia bombi* can cause behavioral changes in infected *Bombus* by affecting their ability to distinguish between flowers with nectar and those without [47]. Consequently, this infection impairs the foraging abilities of bumblebees and reduces their access to food resources. *Crithidia bombi* can also adversely affect the survival rate of bumblebee queens during hibernation and their ability to establish a nesting site (Table 3) [166,167]. Workers infected with *C. bombi* experience decreased body fat, which impairs their immunity and metabolism [168]. Infected queens also exhibit a decreased fat body, which compromises their hibernation survival capacity and prevents them from founding colonies [130,131]. *Ascosphaera apis* was prevalent in the digestive tract of bumblebee larvae and had similar signs to the chalkbrood disease observed in honey bees (Figure 4) [16]. The spores germinate within the larval digestive tract, producing hyphae that invade the tissues and lead to the formation of larval mummies, resulting in a fatal infection. In North American bumblebee queens (*B. griseocollis*, *B. vosnesenskii*, and *B. nevadensis*) infected with *A. apis*, the entire body cavity was observed to be filled with white spongy mycelia [49].

**Table 3:** Pathogens and their impact on bumblebee health.

Pathogen Type	Impact on Bumblebees	References
Viruses	Wing malformations, affects foraging behavior, impairs navigational capacity, and paralysis	[15,157,158,159]
Fungi	Reduced production of viable sperm, infected queens produce fewer offspring, disturbed colony size, decreased lifespan of all stages, causes digestive problems, and impacts immunity	[16,46,161]
Protists	Affects the bumblebee gut, reducing fitness and foraging efficiency, reduced fecundity and lifespan, and decreased body fat	[47,168]

A study examining the incidence and infectivity of the microsporidian parasite *Vairimorpha ceranae* in wild bumblebee species found that the infection decreased bumblebee survival by 48% and discovered a high prevalence of the spores of this parasite in their guts [155]. *Bombus terricola* and numerous other bumblebee species have faced rapid decline across North America in recent years. In this species, five pathogens, namely *Vairimorpha ceranae* (microsporidian parasite), black queen cell virus (RNA viruses), sacbrood virus, and *Lotmaria passim* and *Crithidia bombi* (trypanosomatid parasites), were detected [21]. It was found that the effect of neogregarine *Apicystis bombi* and the deformed wing virus on *B. terrestris* was 22% and 50%, respectively, whereas the combined death rate was 86% [131]. A survey of bumblebees in southern Chile found numerous viral diseases (ABPV, BQCV, and DWV) and pathogens (*Apicystis bombi* and *Crithidia bombi*) in both native (*B. dahlbomii*) and non-native (*B. ruderatus* and *B. terrestris*) species, which caused the decline of their populations [72]. According to a recent investigation in the UK, the acute bee paralysis virus (ABPV) was the most prevalent virus in bumblebees and has caused a significant reduction of the bumblebee population [81].

### 3.3. Interaction Between Pathogens and Environmental Change

**Figure 5:**
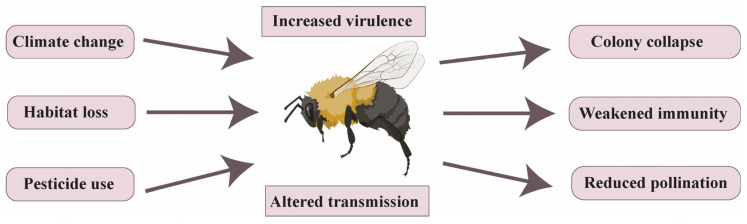
Interaction between environmental changes and pathogen dynamics, and their impacts on bumblebees.

There are multiple stressors that bumblebees often face simultaneously, for example, habitat loss, pesticide exposure, and climate change [169]. All those stressors might interact negatively with one another to have effects on bumblebee health [170] (Figure 5). For example, pesticide exposure suppresses immunological responses and detoxification mechanisms, rendering the bees susceptible to parasites [171]. The exposure of bees to pesticides has been found to have adverse effects on their gut microbiota, leading to an increased vulnerability to infection by opportunistic pathogens [172]. The mortality rate of honey bees was higher when they were subjected to both the insecticide fipronil and concurrent infection with *V. ceranae* compared to instances where just one stressor was present [173]. The compound known as imidacloprid has the potential to have a synergistic effect when combined with *Vairimorpha*, leading to an increase in the occurrence of *Vairimorpha* infections within beehives and subsequently elevating the mortality rate associated with *Vairimorpha* infections [174,175]. The susceptibility of bees to *V. ceranae* is increased as a result of developmental exposure to neonicotinoid pesticides [176].

Climate change is one of the most interconnected factors in pollinator decline [177]. Climate change has direct effects on physiology and morphology and indirect effects through changing abiotic and biotic interactions, such as land use, species competition, invasive insect spread, pathogen susceptibility, and disease emergence [178]. A study shows that the prevalence of *C. bombi* in bumblebees varies throughout the season. The infection was observed in June with low values for *B. terrestris* (14.8%) and *B. lapidarius* (19.0%), rose to its highest in July for *B. terrestris* (77.8%) and *B. lapidarius* (64.6%), and gradually decreased in August for both *B. terrestris* (58.3%) and *B. lapidarius* (62.5%) [143]. The infestation of *V. bombi* was found to have a statistically significant positive correlation with the relative humidity and temperature. *Vairimorpha* spores multiplied more in the higher temperatures and relative humidity in August, increasing the mortality rates among the *B. haemorrhoidalis* queens in field conditions [30]. The incidence of *Vairimorpha* infection was low during winter (October to December), rapidly increased during summer, and gradually increased from March to July [30,161]. Climate change also impacts the interaction between pathogens and bumblebees. Changes in climate, particularly rising temperatures, may result in increased host growth and host density, leading to higher rates of transmission [179]. Similarly, rising temperatures are expected to increase parasite–host infections. Temperature has been assessed as positively correlated with infection in the host *B. terrestris* [180].

The habitat type has a significant impact on the prevalence of pathogens. Losing habitat brings about the reduction of floral resources and satisfactory nesting sites, which impacts the pathogens and their transmission [181]. Sharing resources at extensive levels as the floral resources are limited creates a higher risk of pathogen transmission [182]. The decrease in floral resources leads to reduced nutrition, which has an effect on the bees’ fitness and immunity, rendering the host more at risk of pathogen infection [183,184,185]. As an example, a current study performed on *B. terrestris* found a significantly greater prevalence of *C. bombi* in urban landscapes compared to field vegetation [33].

## 4. Conservation and Management Strategies 

Studies have consistently found the presence of apiaries and honey bee virus loads to be important predictors of bumblebee pathogen prevalence [40,41]. This indicates that beekeeping management standards could be introduced to reduce the import and spread of diseases between bees [82]. All commercial bees should be transported under rigid quarantine protocols for the production of disease-free commercially available bumblebees [186]. The planting of suitable flowers in public areas and gardens can also support conservation regarding pollinators [7]. In addition, the sowing of flower-rich field edges on semi-natural habitats results in a higher abundance and diversity of wild bee populations [186,187].

The immunological responses of bees are influenced by their diet, which indicates that the innate protection against infections of bees can be enhanced by the floral resources consumed by bees [188]. A study showed that the utilization of sunflower pollen (*Helianthus annuus*) reduced the severity of *C. bombi* infection in worker bumblebees (*Bombus impatiens*) [189]. After one week, more than two-thirds of the bees fed sunflower pollen had no detectable infection, and the degree of infection was reduced by 20 to 50 times compared to other pollen diets [188]. Therefore, pollen irradiation protocols (to kill pathogens) could support bumblebee nutrition, improve colony quality, and reduce the risk of pathogen spillover [132].

Pesticide exposure can also have adverse effects on immunological responses and detoxification mechanisms, making bees more vulnerable to pathogen infections [171,172]. Pesticides, including herbicides, insecticides, and fungicides, are applied to crops, roadsides, gardens, and lawns, and bees can collect them while foraging [190]. To reduce bee poisonings, sustainable bee management practices can be used by avoiding the use of common pesticides, especially pyrethroids and neonicotinoids [191]. Farmers and homeowners can also make simple changes to help pollinators, such as avoiding treatments near blooming flowers, near nesting areas, and during the active time of bees [192].

Some methodological approaches should also be implemented at a large scale to prevent the damage to wild pollinators caused by pathogens. As these approaches have been implemented in the UK, the Bumblebee Conservation Trust (BCT) provides protocols for managing and preventing diseases in bumblebee colonies [193]. This results in healthier populations of wild pollinators. Similarly, habitat-focused strategies were implemented in the UK’s Countryside Stewardship Scheme, where flower-plant-rich fields were planted around the margins of fields to enhance the abundance and richness of wild pollinators [194]. Finally, a good diet is also suggested to reduce the impact of pathogens; sunflower pollen is already used to reduce *C. bombi* in bumblebee species [189].

## 5. Conclusions

Bumblebee species are highly significant pollinators for a wide range of crops, fruits, and vegetables. Nevertheless, the populations of these species have been experiencing a significant decline at unprecedented rates, resulting in substantial ecological and economic consequences. Bumblebees face numerous biotic and abiotic threats that they must protect themselves from in order to survive. Among the biotic factors, pathogens are frequently cited as major causes of population declines in both managed and wild bee species. This review article has elucidated the crucial role of pathogens in the reduction of bumblebee species populations. A diverse range of pathogens affects bumblebees, including viruses, trypanosomes, neogregarinida, and microsporidia. These pathogens have the ability to cause significant effects on individual bumblebees as well as entire colonies, resulting in increased mortality rates and decreased reproductive potential. Worker bumblebees are found to be more susceptible to viral infections because they may come into contact with honey bees’ viruses while visiting the same flowers. There are concerns regarding the spread of pathogens from managed bumblebees to wild bee populations, which has required the development of effective management strategies against bumblebee pathogens. Bumblebees’ susceptibility to pathogen infections is enhanced by stressors like climate change, habitat loss, and pesticide exposure. Clearly, a holistic approach is required to address the multiple stressors faced by bumblebees, with a focus on pathogen and disease management. Pollinator-friendly environments are necessary to protect bumblebees and their vital role in biodiversity and food security through public awareness, policy interventions, and global cooperation.

## Data Availability

The original contributions presented in the study are included in this article; further inquiries can be directed to the corresponding author.
